# Biogeography and diversity patterns of abundant and rare bacterial communities in paddy soils along middle and lower Yangtze River

**DOI:** 10.1002/ece3.11481

**Published:** 2024-06-04

**Authors:** Xiancan Zhu, Minghui Hu, Xiaoli Wang, Ya Zhang, Dongsheng Du

**Affiliations:** ^1^ Collaborative Innovation Center of Recovery and Reconstruction of Degraded Ecosystem in Wanjiang Basin Co‐Founded by Anhui Province and Ministry of Education Anhui Normal University Wuhu China; ^2^ Anhui Provincial Key Laboratory of Molecular Enzymology and Mechanism of Major Metabolic Diseases, College of Life Sciences Anhui Normal University Wuhu China

**Keywords:** agricultural ecosystem, biogeography, ecological processes, microbial community, rice field, soil bacterial diversity

## Abstract

The middle and lower reaches of the Yangtze River serve as principal rice production bases in China, yet the biodiversity and ecological processes of bacterial communities in paddy soils are not well understood. This study explores the diversity, composition, ecological function, and assembly processes of abundant and rare bacterial communities in paddy soils. A total of 129 paddy soil samples from 43 sites along the middle and lower reaches of the Yangtze River were collected and analyzed using NovaSeq sequencing. The results showed that the dominant phylum for both abundant and rare taxa was Proteobacteria, with a greater relative abundance of the abundant taxa. The diversity of the abundant community was lower than that of the rare community. Soil properties and geographic variables explained more of the variation in the abundant community than in the rare community. The rare community exhibited a significant distance‐decay relationship. The assembly of the abundant community was more influenced by stochastic processes, although both the abundant and rare communities were governed by stochastic processes. It is concluded that both abundant and rare bacterial communities exhibit differing biogeographic patterns, yet they undergo similar ecological processes in the paddy soils along the middle and lower reaches of the Yangtze River. These observations offer a theoretical framework for a deeper comprehension of the function of both abundant and rare bacteria, as well as the development and preservation of soil bacterial diversity within agricultural ecosystems.

## Graphical abstract

Abundant and rare bacterial communities exhibited differing biogeographic patterns, and Proteobacteria was the dominant phylum for both abundant and rare taxa in the paddy soils along the middle and lower of the Yangtze River. The assembly of the abundant community was more influenced by stochastic processes, although both the abundant and rare communities were governed by stochastic processes.

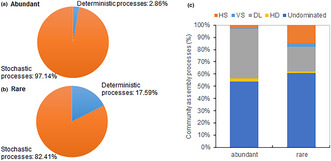

## 1 INTRODUCTION

Rice stands as a principal food crop globally, with China being the top producer and consumer. Paddy soils are vital components of agricultural soil ecosystems, significantly impacting food security and terrestrial ecosystem functions (Hou et al., 2020; Zhang et al., 2024). Soil microorganisms are essential in agricultural ecosystems, playing pivotal roles in soil processes and agricultural productivity. These include nutrient uptake and cycling, turnover of soil organic matter (SOM), transformations and sequestration of soil carbon, soil structure, greenhouse gas emissions, and enhancement of crop stress resistance and yield (Banerjee & van der Heijden, 2023; Hartmann & Six, 2023). Exploring the diversity and biogeographic patterns of soil microbes is crucial to understanding the ecological processes and mechanisms that support and sustain ecosystem functions (Hanson et al., 2012). Consequently, grasping the biogeography and diversity of microbes in paddy soils is essential for assessing soil health and function within agricultural ecosystems.

Soil microorganisms in natural ecosystems consist predominantly of a multitude of low‐abundance taxa and a few high‐abundance taxa, categorized respectively as rare and abundant groups (Lynch & Neufeld, 2015; Zhou et al., 2022). Recently, there has been an increasing focus on the ecological significance of these abundant and rare communities. They often display varied distribution patterns, turnover rates, and functional traits in response to environmental disturbances (Liu et al., 2023). Thus, delineating the differences between abundant and rare taxa is crucial for understanding the processes and functions of microbial communities in ecosystems (Jiao & Lu, 2020a). Previous studies have demonstrated differences in the biogeography, composition, and potential functions of abundant and rare microbial communities in various ecosystems, such as forests (He et al., 2023), farmlands (Chang et al., 2022), lakes (Ren et al., 2022), and deserts (Wang et al., 2021). In agricultural soils, for example, Zhou et al. (2022) reported that the diversity of soil bacterial communities differed between abundant and rare subcommunities and that the potential functions of rare taxa were greater than those of abundant taxa in farmlands. Compared with abundant taxa, rare taxa have been found to have greater metabolic activity and are likely to regulate ecosystem functions as key taxa (Lynch & Neufeld, 2015). However, the distinct ecological mechanisms of abundant and rare communities in agricultural ecosystems are still not well understood.

It is widely acknowledged that both stochastic processes (such as dispersal limitation, homogenizing dispersal, and drift) and deterministic processes (including variable selection and homogeneous selection through environmental filtering, and biotic interactions) are fundamental in governing microbial community assembly (Kang et al., 2022; Li et al., 2022; Xun et al., 2019). There is mounting evidence that both abundant and rare communities are shaped by stochastic and deterministic processes (He et al., 2023; Ji et al., 2020; Xu et al., 2022). Yet, the relative significance of these two ecological processes in influencing these communities remains a subject of debate. Mo et al. (2018) observed that dispersal was more limited for rare bacterial communities than for abundant communities across three subtropical bays in China. Conversely, Wan et al. (2021) identified that dispersal limitation was predominant in the assembly of abundant bacterial communities, while variable selection was more decisive for rare communities in wetland soils of the Qinghai‐Tibet Plateau. These divergent findings may be attributed to variations in geographic scales, environmental gradients, and habitat conditions (Hanson et al., 2012; He et al., 2023).

The Yangtze River is the longest river in China and runs through central China from west to east. Its middle and lower reaches are the regions with the highest population density and the most severe environmental pressure, and they are the main rice production bases in China. However, the biogeographic pattern, ecological function, and assembly processes of abundant and rare bacterial communities in agricultural soils of the Yangtze River remain unclear. In this study, we investigated the soil bacterial community diversity and structure in rice fields along the middle and lower reaches of the Yangtze River. The differences in the biogeographical patterns and community assemblies of the abundant and rare communities and the soil properties influencing the abundant and rare communities in paddy soils along the river were also analyzed.

## 2 MATERIALS AND METHODS

### 2.1 Soil sampling

Soil samples were collected from paddy fields along the middle and lower reaches of the Yangtze River, spanning eastward from Yichang City in Hubei province to the East China Sea, covering a distance of 1893 km and an area of 0.8 million km^2^. This region features a subtropical monsoon climate, with average annual temperatures ranging from 14 to 18°C and annual precipitation of about 1000 mm, favorable for agricultural production. A total of 129 paddy soil samples from 43 sites were collected between September 27 and October 14, 2020 (Zhang et al., 2024). At each site, five soil cores (5.5 cm in diameter and 20 cm deep) were taken and combined to form a composite sample. From each site, three plots of 100 m^2^ were chosen for sampling. The collected soil samples were transported to the laboratory, where they were sifted through a 2 mm sieve to eliminate plant debris and stones and then divided into two parts. One part was preserved in liquid nitrogen, freeze‐dried using a vacuum freeze drier, and stored at −80°C for DNA extraction, while the other was used for soil property analysis after air drying.

### 2.2 Soil properties assays

Soil chemical properties, including soil pH, soil organic carbon (SOC), total nitrogen (TN), ammonium nitrogen (NH_4_^+^‐N), nitrate nitrogen (NO_3_^−^‐N), and Olsen‐P, were quantified using standard test methods (Lin, 2004). Activities of soil urease, phosphatase, and invertase were assessed using the sodium phenol‐sodium hypochlorite method, disodium diphenyl phosphate method, and 3,5‐dinitrosalicylic acid method, respectively (Zhu et al., 2018).

### 2.3 DNA extraction and sequencing

Soil DNA was extracted using the DNeasy Power Soil Kit (Qiagen, Hilden, Germany) following the manufacturer's protocol. The quality and concentration of the extracted DNA were evaluated using 1.2% agarose gel electrophoresis (DYY‐6C, Liuyi, Beijing, China) and an ultraviolet spectrophotometer (Nanodrop NC2000, Thermo Scientific, Waltham, MA, USA). Amplification of the 16S rRNA V3‐V4 region was performed via PCR with the primers 338F (ACTCCTACGGGAGGCAGCA) and 806R (GGACTACHVGGGTWTCTAAT) (Yang et al., 2020). The 25 μL PCR mixture included a DNA template, primers, dNTPs, reaction buffer, and Q5 high‐fidelity DNA polymerase (NEB, Ipswich, MA, USA). The PCR protocol involved an initial denaturation at 98°C for 2 min, 25 cycles of 15 s at 98°C, 30 s at 55°C, and 30 s at 72°C, and a final extension at 72°C for 5 min. The fluorescence of the PCR products was measured using the Quant‐iT PicoGreen dsDNA Assay Kit (Invitrogen, Camarillo, CA, USA) after preliminary electrophoresis. The DNA samples were sequenced on the Illumina NovaSeq 6000 system (Illumina, San Diego, CA, USA) with the NovaSeq 6000 S4 Reagent Kit V1.5 (paired‐end, 300 cycles, 2 × 250 bp) at Personal Biotechnology Co., Ltd. (Shanghai, China), and the sequence data were uploaded to the NCBI Sequence Read Archive (project ID, PRJNA822031).

### 2.4 Bioinformatics and data analysis

Microbiome bioinformatics analyses were performed using QIIME 22019.4 (Bolyen et al., 2019) with some modifications. In brief, the raw sequence data were demultiplexed using the demux plugin, and the primers were removed using the cutadapt plugin. The sequences were then subjected to quality filtering, denoising, and merging with chimera removal via the DADA2 plugin (Callahan et al., 2016). Subsequently, a total of 217,131 non‐singleton amplicon sequence variants (ASVs) were aligned using MAFFT and utilized to construct the phylogeny with FastTree2. Taxonomies were assigned to the ASVs using the classify‐sklearn naive Bayes taxonomy classifier and the Silva v132 99% operational taxonomic units reference sequences (Quast et al., 2013).

ASVs with relative abundance lower than 0.01% of the total sequences were defined as rare ASVs, while ASVs with relative abundance higher than 0.1% were defined as abundant ASVs (Jiao & Lu, 2020b). The alpha diversity of bacteria was evaluated by the Observed species and Shannon index, which indicate the richness and diversity of the ASV community, respectively. The structure of the bacterial communities was assessed using non‐metric multidimensional scaling (NMDS) based on the Bray‐Curtis distance. The potential functions of the bacterial communities were predicted using Phylogenetic Investigation of Communities by Reconstruction of Unobserved States (PICRUSt2) and annotated with the Kyoto Encyclopedia of Genes and Genomes (KEGG) function (Langille et al., 2013).

Statistical analyses were conducted using IBM SPSS22.0 (SPSS Inc., Armonk, NY, USA) along with the “vegan” and “ggplot2” packages in the R environment (v4.1.0). Data were logarithmically transformed prior to analysis to approximate a normal distribution as closely as possible. When variables failed to meet ANOVA assumptions, the non‐parametric Kruskal‐Wallis test was implemented. For post‐hoc multiple comparisons of means exhibiting distinct effects, Duncan's test was used. Statistical significance was established at *p* < .05. Spearman correlation analysis identified the correlation coefficients among bacterial communities (both abundant and rare), as well as geographic and soil property variables. The Mantel tests, involving 999 permutations, assessed the relationships between the taxonomic and functional compositions of these communities and geographic and soil property variables using the “linkET” R package. The “vegan” package's Adonis function conducted Permutational Multivariate ANOVA (PERMANOVA) with 999 permutations to identify significant variables. Canonical correspondence analysis (CCA) explored the relationships between community types and environmental variables using the “vegan” package, with significant variables identified via the “envfit” function after 999 permutations. Variation partition analysis (VPA) was performed with the “varpart” function in “vegan” to ascertain the impacts of geographic and soil properties on community structures. Distance‐decay relationships were quantified by geographical distance and community similarity, with distance‐decay slopes calculated through least‐squares regression. Lastly, the assembly of abundant and rare communities' ecological processes was examined using a phylogenetic‐bin‐based null model analysis in the “iCAMP” R package (Ning et al., 2020).

## 3 RESULTS

### 3.1 Relative abundances of abundant and rare taxa

The abundant taxa comprised 31 ASVs distributed among 7 phyla, 13 classes, 21 orders, 22 families, and 25 genera. In contrast, the rare taxa included 124,863 ASVs distributed among 59 phyla, 176 classes, 468 orders, 840 families, and 2017 genera.

**FIGURE 1:**
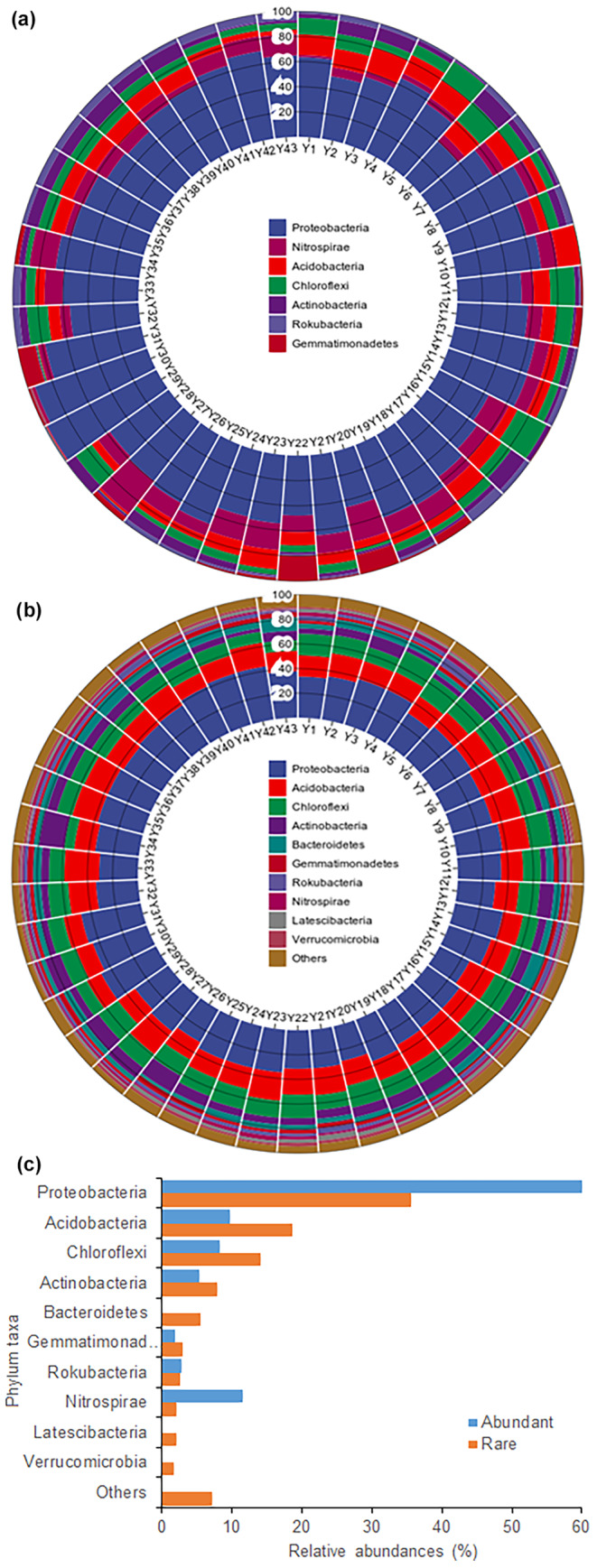
Relative abundances of bacterial predominant phyla of abundant (a) and rare (b) taxa at 43 sampling sites, and average relative abundances of abundant and rare taxa (c).

At the phylum level, the predominant bacteria in abundant taxa were Proteobacteria, Nitrospirae, Acidobacteria, Chloroflexi, Actinobacteria, Rokubacteria, and Gemmatimonadetes, with average relative abundances of 60.59%, 11.59%, 9.72%, 8.22%, 5.26%, 2.82%, and 1.81%, respectively (Figure 1a). Conversely, the main phyla of rare taxa exhibited relative abundances of Proteobacteria, Acidobacteria, Chloroflexi, Actinobacteria, Bacteroidetes, Gemmatimonadetes, Rokubacteria, Nitrospirae, Latescibacteria, and Verrucomicrobia at 35.66%, 18.62%, 14.02%, 7.82%, 5.49%, 2.92%, 2.63%, 2.03%, 1.95%, and 1.65%, respectively (Figure 1b). Notably, Proteobacteria emerged as the dominant phylum in both abundant and rare taxa, albeit with varying relative abundances (Figure 1c).

**FIGURE 2:**
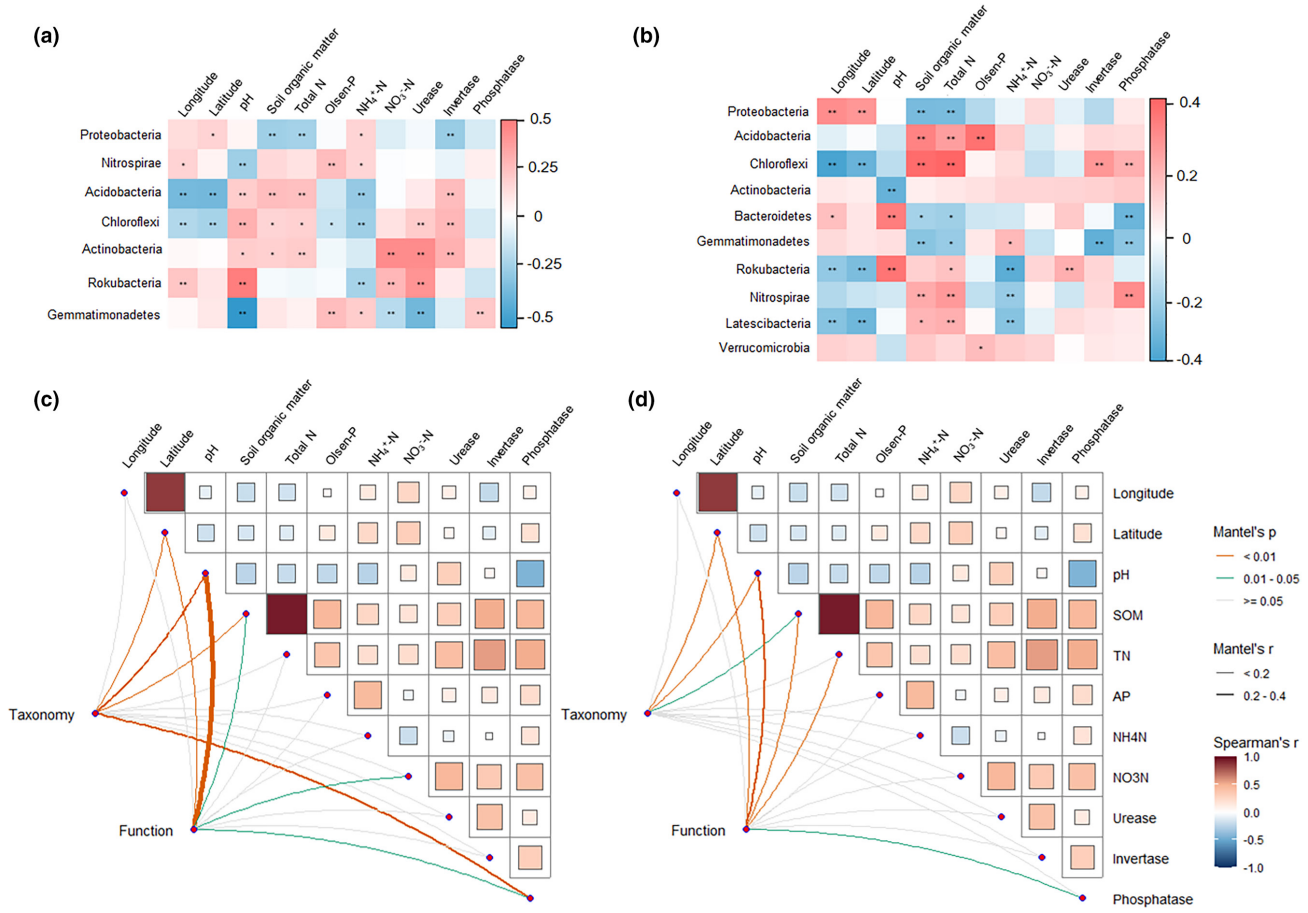
Relationships of relative abundances of predominant phyla of abundant (a) and rare (b) taxa with geographic and soil variables. Compositions of taxonomic and functional abundant (c) and rare (d) communities were associated with geographic and soil variables by partial Mantel tests. Pairwise comparisons of geographic and soil variables, and color gradient denoted coefficients of Spearman's correlation.

The relationships between the relative abundance of phyla and geographic as well as soil variables were illustrated in Figure 2a,b. However, there were distinctions in the findings for abundant and rare taxa. For example, the relative abundance of dominant Proteobacteria in abundant bacteria correlated with latitude, SOM, total N, NH_4_^+^‐N, and invertase, while in rare taxa, it showed correlations with longitude, latitude, SOM, and total N. Moreover, Mantel tests revealed that the taxonomic composition of the abundant subcommunities was significantly associated with latitude, pH, SOM, and phosphatase, while the taxonomic composition of the rare subcommunities was associated with latitude, pH, and SOM (Figure 2c,d).

The relative abundances of the top 10 most abundant and rare bacteria at the class, order, family, and genus levels are shown in Appendix S1: Figure S1. The dominant class, order, family, and genus of abundant taxa were Gammaproteobacteria (with a relative abundance of 32.55%), Betaproteobacteriales (17.67%), Nitrosomonadaceae (14.51%), and *MND1* (11.70%), respectively. Conversely, the dominant class, order, family, and genus of rare taxa were Deltaproteobacteria (16.35%), Betaproteobacteriales (9.17%), Subgroup_6 (6.76%), and *Subgroup_6* (6.76%), respectively. Variances in the correlation between the relative abundance of abundant and rare classes, orders, families, and genera with geographic and soil variables were noted (see Appendix S1: Figure S2).

### 3.2 Diversities of abundant and rare communities

**FIGURE 3:**
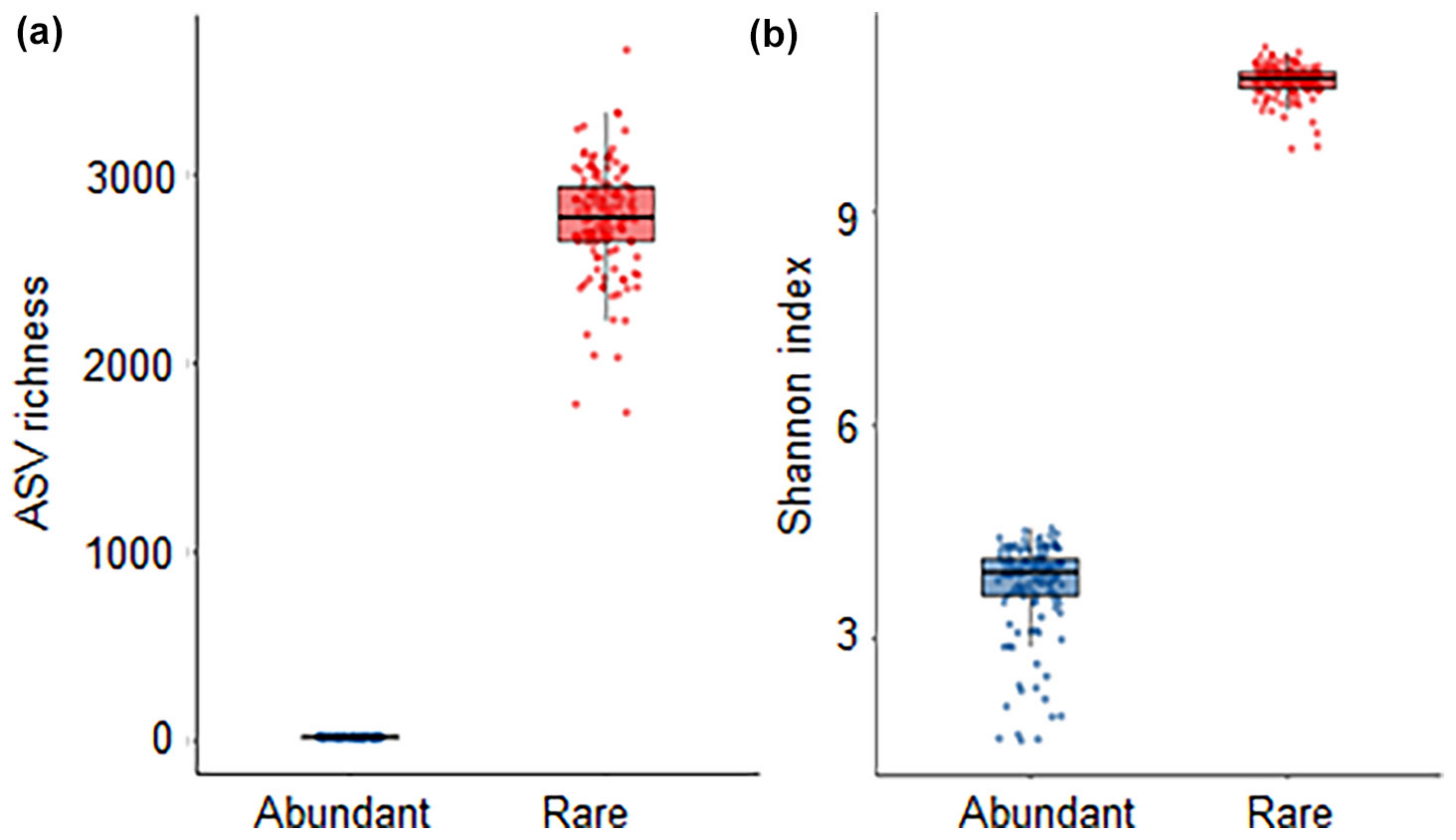
Average values of ASVs richness and Shannon index of abundant and rare bacterial communities.

Compared with the abundant communities, the rare bacterial communities had greater ASV richness and Shannon index (Figure 3). The ASV richness of the abundant and rare communities in paddy soils along the middle and lower reaches of the Yangtze River ranged from 9.33 to 27.53 and 2142 to 3200, respectively (see Appendix S1: Table S1). Similarly, the Shannon index of the abundant and rare species ranged from 1.73 to 4.43 and 10.29 to 11.16, respectively. Furthermore, the ASV richness of the abundant communities was significantly positively correlated with soil pH, SOM, total N, P, and urease and invertase activities, but was negatively correlated with latitude (see Appendix S1: Table S2). The ASV richness of rare communities exhibited a positive correlation with longitude, SOM, total N, NO_3_^−^‐N, and phosphatase activity. Meanwhile, the Shannon index of abundant communities showed a significant correlation with soil pH, SOM, total N, NO_3_^−^‐N, urease, and invertase activities.

Abundant communities exhibited distinct β‐diversity compared to rare communities as observed in the PCoA (see Appendix S1: Figure S3). The PCoA1 scores of abundant communities showed significant association with soil pH, NH_4_^+^‐N, NO_3_^−^‐N, and urease and invertase activities, whereas those of rare communities were associated with pH, SOM, total N, NH_4_^+^‐N, NO_3_^−^‐N, and urease and invertase activities (see Appendix S1: Table S3). PCoA2 scores of abundant communities were significantly correlated with longitude, latitude, pH, SOM, total N, and urease and invertase activities, while those of rare communities were correlated with longitude, SOM, total N, NH_4_^+^‐N, NO_3_^−^‐N, and invertase and phosphatase activities.

### 3.3 Effects of geography and soil properties on abundant and rare community structure

**FIGURE 4:**
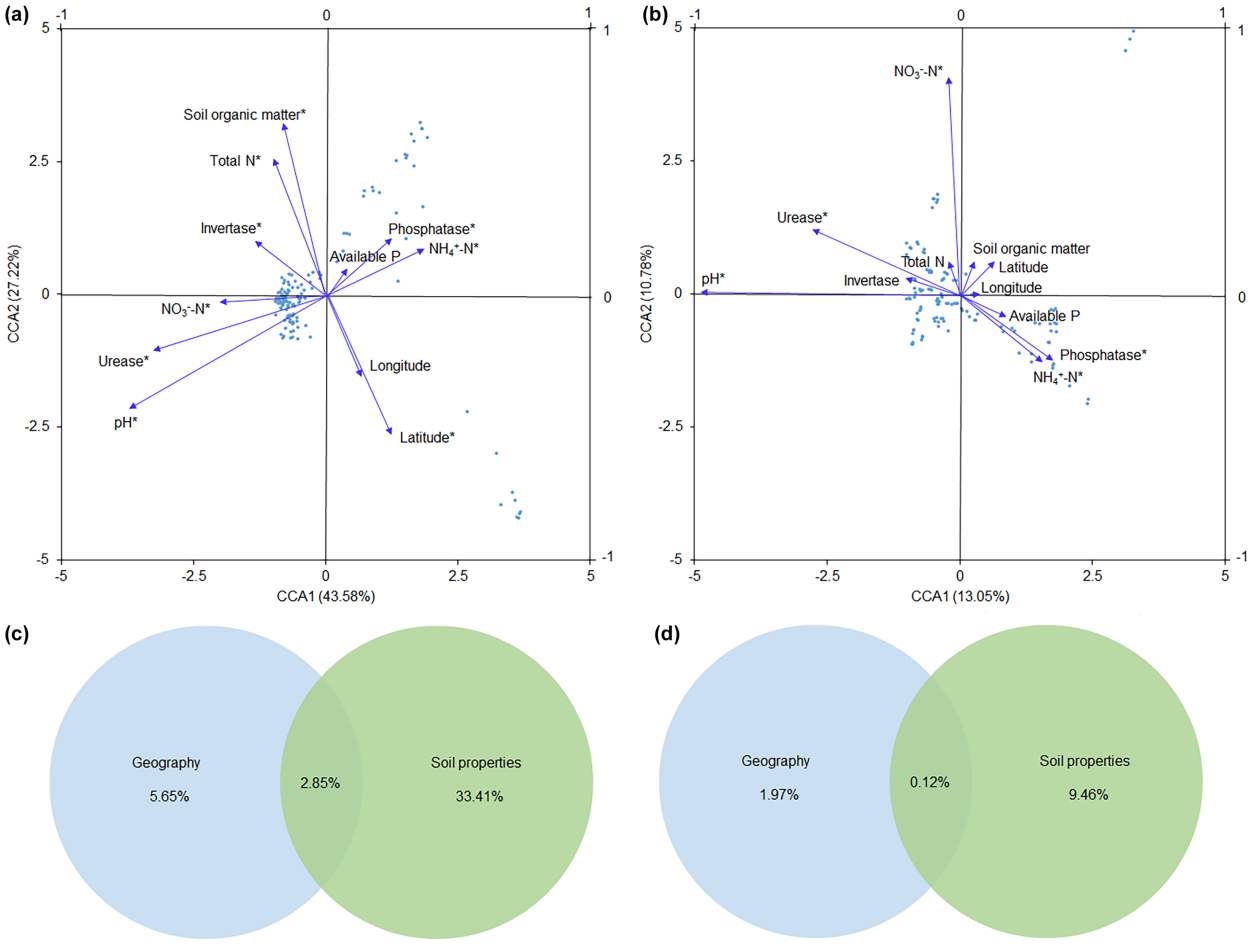
CCA on structures of abundant (a) and rare (b) communities and geographic and soil variables. Variance partitioning presenting abundant (c) and rare (d) community structure variation proportion interpreted by geography and soil properties. *Represents a statistical significance with *p* < .05.

CCA results indicated that the most influential factor shaping abundant community structure was soil pH, followed by urease activity, SOM, latitude, total N, NH_4_^+^‐N, NO_3_^−^‐N, and phosphatase and invertase activities (Figure 4a). Similarly, soil pH was the primary factor affecting rare community structure, followed by NO_3_^−^‐N, urease, phosphatase activities, and NH_4_^+^‐N (Figure 4b). Additionally, VPA demonstrated that soil properties consistently explained more variation than geographic variables for both abundant and rare communities (Figure 4c,d). The combination of geographic and soil variables contributed to a higher interpretation rate in abundant communities (41.91%) compared to rare communities (11.55%).

**FIGURE 5:**
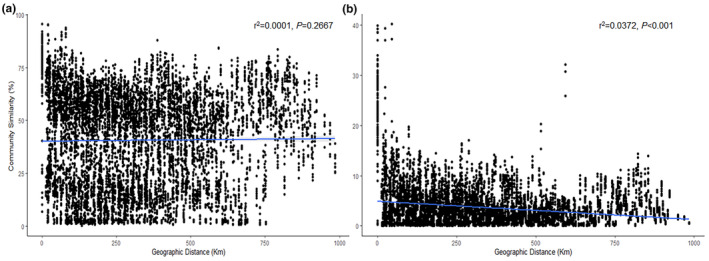
Relationship between abundant (a) and rare (b) community similarity and geographic distance.

A notable distance‐decay relationship was observed in rare communities (Figure 5b). The distance‐decay slope and fitness values were higher in rare communities compared to abundant communities (Figure 5).

### 3.4 Functions of abundant and rare communities

**FIGURE 6:**
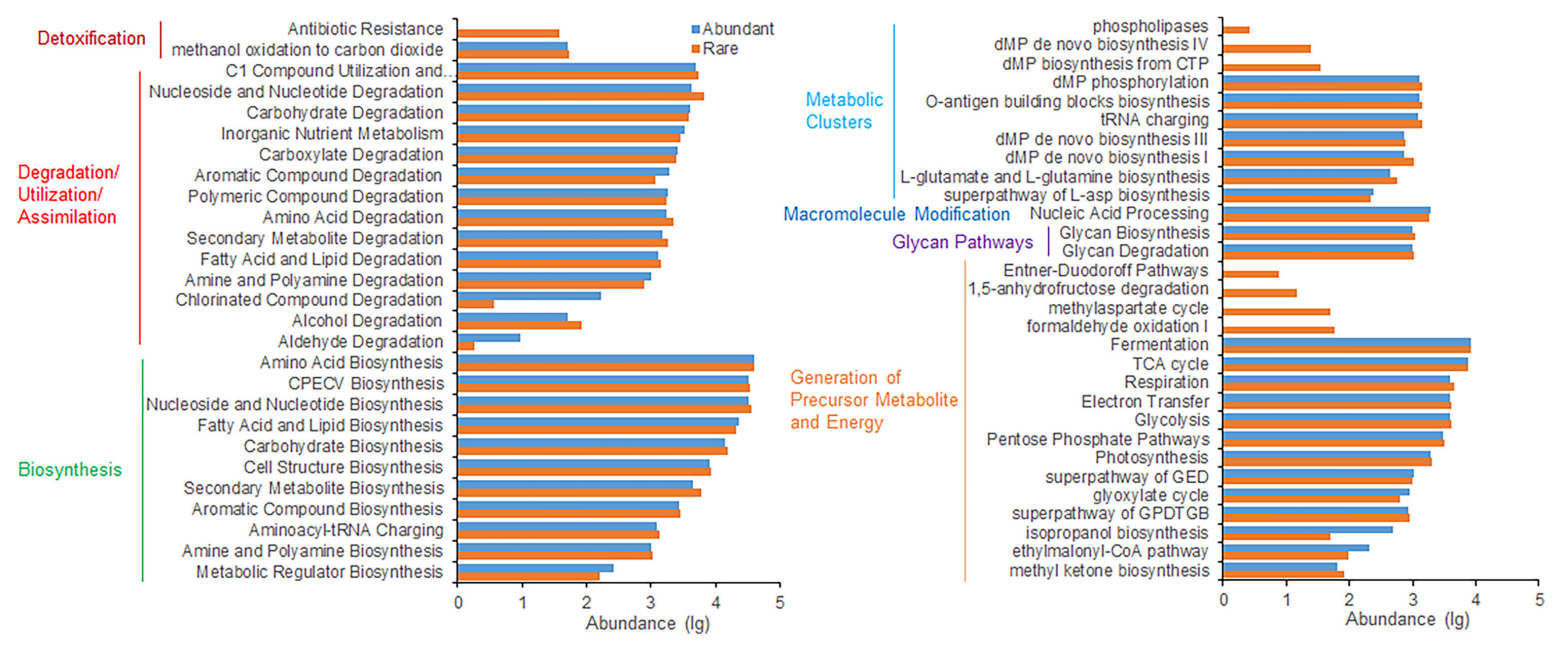
Predicted KEGG functional abundances of abundant and rare communities.

Predictions of potential functions for abundant and rare communities unveiled differences in the relative abundance of KEGG metabolism pathways between them, as depicted in Figure 6. Compared to abundant communities, rare communities showed enrichment in six functions: biosynthesis, degradation/utilization/assimilation, detoxification, generation of precursor metabolite and energy, glycan pathways, and metabolic clusters. Additionally, the relationships between KEGG functional abundances of abundant communities and geographic and soil variables differed from those of rare communities (see Appendix S1: Figure S4). The correlation between the PCoA scores of community functional structure and geographic and soil variables also differed for abundant and rare bacteria (see Appendix S1: Table S4). Mantel tests revealed that the functional composition of the abundant communities was significantly associated with latitude, pH, SOM, urease, and phosphatase, while that of the rare communities was associated with latitude, pH, SOM, total N, and phosphatase activity (Figure 2c,d).

### 3.5 Assembly of abundant and rare communities

**FIGURE 7:**
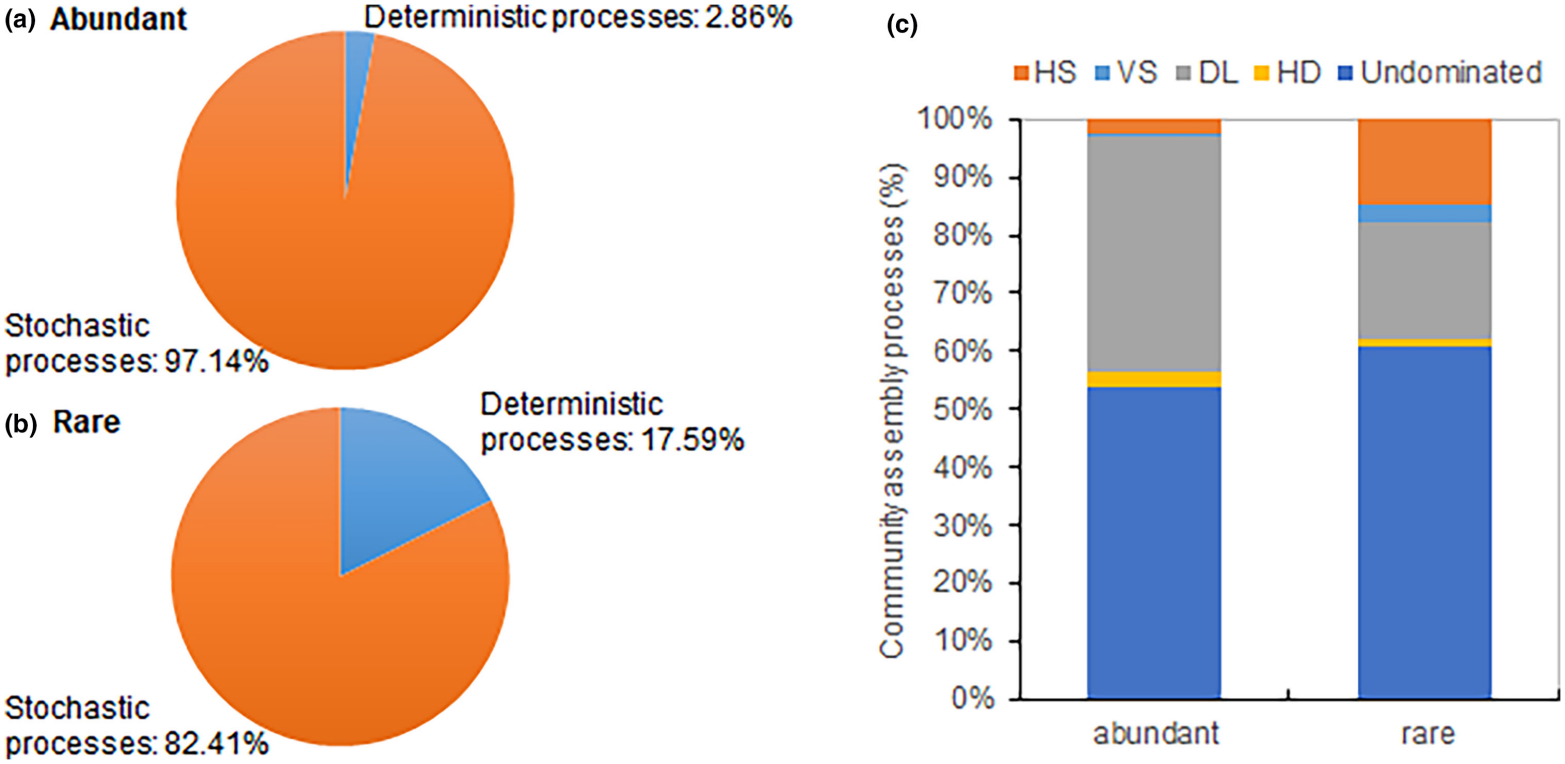
Contribution proportion of stochastic and deterministic processes in abundant (a) and rare (b) community assembly, and variable selection, homogeneous selection, dispersal limitation, homogenizing dispersal, and undominated fraction (c) by the null model analysis.

The analysis based on the phylogenetic‐bin‐based null model revealed that both abundant and rare community assemblies were primarily driven by stochastic processes (Figure 7). Stochastic processes dominated in abundant communities and accounted for 97.14%, while deterministic processes contributed only 2.86%. In contrast, stochastic processes accounted for 82.41% of rare communities, with deterministic processes explaining 17.59%. Within stochastic processes, dispersal limitation and homogenizing dispersal played roles in both abundant and rare communities, with slightly higher proportions in abundant communities, respectively. As for deterministic processes, variable selection and homogeneous selection were more pronounced in rare communities compared to abundant communities.

## 4 DISCUSSION

In this study, our findings showed that abundant and rare communities had distinct biogeographical patterns and ecological functions in paddy soils along the middle and lower reaches of the Yangtze River. This finding was consistent with previous studies conducted in various ecosystems, including agricultural (Zhou et al., 2022), desert (Wang et al., 2021), forest (He et al., 2023), wetland (Wan et al., 2021), and grassland (Ji et al., 2020) ecosystems. Moreover, our study also revealed that abundant taxa exhibit similar community assembly compared with rare taxa.

Previous studies have highlighted varied ecological processes influencing abundant and rare communities across diverse environments (He et al., 2023; Wan et al., 2021). In our study, null model analysis indicated that both abundant and rare community assemblies were shaped by stochastic processes, potentially attributed to ongoing flooding management practices and regular dry‐wet cycles in rice paddy soils, leading to reduced environmental filtering (Hu et al., 2013; Jiao & Lu, 2020b). Ren et al. (2022) similarly demonstrated that stochastic processes governed both abundant and rare bacterial communities. However, the relative importance of stochastic and deterministic processes in abundant and rare community assembly remains debated due to variations in geographic scales, habitat conditions, and environmental perturbations (He et al., 2023; Shi et al., 2018; Wang et al., 2017). For instance, Hou et al. (2020) found that deterministic selection was the primary assembly process for abundant communities, while stochastic processes governed rare community assembly in rice paddy soils across China. Nonetheless, Jiao and Lu (2020b) discovered that homogeneous selection processes dominated the assembly of both abundant and rare communities in maize and rice fields across eastern China, indicating that the assembly of abundant and rare bacterial communities depends on distinct environmental variables.

In our study, stochastic assembly processes were more prevalent in abundant communities than in rare communities, which contrasts with previous studies indicating a stronger influence of stochastic processes on rare community assembly in rice paddy soils (Hou et al., 2020; Jiao & Lu, 2020b). The discrepancy in the impact of stochasticity on abundant and rare communities may stem from their differing life strategies (Ji et al., 2020). Additionally, our study revealed a greater influence of dispersal on abundant communities compared to rare communities. Wu et al. (2017) similarly observed a higher degree of dispersal limitation in the abundant community compared to the rare community in the surface layer of the north‐western Pacific Ocean. The limited dispersal ability of rare taxa, resulting from their small population sizes and narrow niche breadths, may contribute to this pattern (Jousset et al., 2017). Taxa with broad niche breadths (habitat generalists) may be more influenced by dispersal limitation, while those with narrow niche breadths (habitat specialists) may be more governed by environmental selection (Pandit et al., 2009; Ren et al., 2022). Our study further supports this idea by demonstrating that rare communities are more affected by environmental selection than abundant communities.

Soil microbial community diversity and composition were driven by environmental conditions (Hartmann & Six, 2023; Zhu et al., 2018). In this study, we found that soil pH, SOM, N, P, and enzyme activities influenced soil abundant and rare community diversity and composition. However, abundant and rare communities exhibited differential responses to soil properties. Generally, soil properties had a stronger association with abundant community diversity and composition than with rare community diversity and composition, suggesting that abundant communities were more dependent on soil properties. This finding was further supported by VPA, which indicated that soil properties explained a greater proportion of the variation in abundant community composition than in rare community composition.

Soil pH is widely recognized as a key driver influencing the diversity and composition of soil bacterial communities (Du et al., 2023; Jiao & Lu, 2020b). In this study, pH was significantly associated with both abundant and rare community composition, indicating that pH was a determining factor in regulating community composition. However, pH was significantly correlated with abundant diversity, but not with rare diversity. Jiao and Lu (2020b) reported that soil pH had the strongest effect on the abundant community structure in agricultural fields across eastern China. Hou et al. (2020) suggested that soil pH had a quadratic association with both abundant and rare community diversity, with more rare species living in acidic and alkaline soils. Previous studies have also shown that abundant bacteria exhibit strong tolerance to saline and alkaline soils (Delgado‐Baquerizo et al., 2018; Ji et al., 2020). The intracellular pH of bacteria can influence membrane‐bound proton pumps and protein stability, resulting in a relatively narrow growth tolerance to pH (He et al., 2023; Wu et al., 2019).

In the present study, we observed differential effects of soil nutrients on various bacterial subcommunities. Soil bacterial community diversity and composition are significantly influenced by the availability and balance of nutrients. For example, SOM positively correlated with both abundant and rare community diversity, indicating that high SOM content can support complex bacterial communities with greater diversity (Garrido‐Benavent et al., 2020; Ren & Gao, 2022). Prior research consistently highlights that abundant taxa often possess broader environmental adaptations, higher nutrient utilization potential, and greater competitive ability (Wan et al., 2021; Zhalnina et al., 2018). In contrast, rare taxa tend to occupy narrower niches, exhibit lower resilience or resistance, and have slower growth rates, rendering them less adaptable to environmental changes compared to abundant taxa (Pascoal et al., 2021; Reveillaud et al., 2014). Additionally, He et al. (2023) proposed that the broader environmental tolerance of abundant taxa may stem from their enhanced dispersal ability and more effective resource acquisition compared to rare taxa. Notably, a significant proportion of the variation in both abundant and rare communities remained unexplained in our study, likely due to unexamined abiotic and biotic factors (Hanson et al., 2012). Therefore, future work should consider incorporating additional ecological variables to comprehensively explore the structure of soil microbial communities.

In this study, our findings unveiled disparities in the spatial distribution patterns of soil bacteria between abundant and rare communities in paddy fields. We observed a significant negative correlation between latitude and abundant community diversity. Moreover, CCA revealed a significant correlation between abundant community composition and latitude. However, no significant correlation was observed between latitude and rare community diversity and composition. These results indicated that latitude, as a crucial spatial variable, influences the distribution and structure of abundant communities at the regional scale. Furthermore, we observed a distance‐decay relationship in the rare communities, with a steeper slope of the distance‐decay curve than in the abundant communities. This finding is consistent with previous studies in paddy soils (Hou et al., 2020) and temperate desert ecosystems (Wang et al., 2021), indicating a greater turnover rate of rare bacteria than of abundant taxa. Both dispersal limitation and environmental selection are the main processes contributing to distance‐decay relationships (Hanson et al., 2012; Lear et al., 2014). Therefore, the weaker distance‐decay relationships observed in our study may be due to human disturbance, habitat differences, limited dispersal, and/or environmental adaptability (Clark et al., 2021; Wang et al., 2017; Zhou et al., 2022). Unexpectedly, rare species were less impacted by geography than abundant species in the VPA plot, which may be due to a greater proportion of unexplained variation in the rare community composition than in the abundant community.

In the present study, the abundant taxa exhibited fewer taxonomic groups compared to the rare taxa, consistent with findings from numerous previous studies (Jiao & Lu, 2020b; Liu et al., 2023; Ren et al., 2022). Our results highlighted Proteobacteria and Nitrospirae as the dominant phyla among abundant taxa, whereas Proteobacteria and Acidobacteria were prevalent among rare taxa. Hou et al. (2020) also noted that Proteobacteria is the most abundant bacterial phylum in rice paddy soils across China, with substantial compositional differences between abundant and rare subcommunities. Similarly, Zhou et al. (2022) observed the dominance of Actinobacteria and Proteobacteria in abundant bacterial subcommunities, while Proteobacteria and Chloroflexi dominated the rare subcommunities in dryland farmland in northeastern China. Proteobacteria, being copiotrophic bacteria, are widely distributed in agricultural systems and are adapted to high nutrient conditions (Dai et al., 2018; Zhu et al., 2018). Nitrospirae, known for its broad habitat range, has been identified in various terrestrial and aquatic ecosystems (Meng et al., 2023). As aerobic chemolithoautotrophic nitrite‐oxidizing bacteria, Nitrospirae plays a pivotal role in nitrification and other processes crucial to the carbon, nitrogen, and sulfur cycles in environments (Daims & Wagner, 2018; Meng et al., 2023).

It is widely acknowledged that abundant and rare communities serve distinct functions across various ecosystems (Ren et al., 2022; Xue et al., 2020). Abundant taxa are recognized as pivotal in regulating ecosystem functioning (Pedrosalio, 2012). However, mounting evidence underscores the significance of rare taxa in mediating ecosystem stability and function (Lynch & Neufeld, 2015; Xue et al., 2020). In our study, we observed differences in predicted KEGG functions between abundant and rare communities, with rare communities enriching more functions than abundant communities, consistent with findings by Zhou et al. (2022), who reported that rare Proteobacteria harbored more potential functions compared to abundant taxa. Previous study has also demonstrated that the disappearance of rare taxa is associated with the loss of key specialized functions in soils (Singh et al., 2014). Moreover, our results indicated that both abundant and rare communities were correlated with SOM, N, and soil enzyme activities, suggesting that rare communities play an irreplaceable role in maintaining ecosystem functions such as carbon and nutrient cycling (Pester et al., 2010; Xue et al., 2020).

In summary, our study provides insights into the biogeography and assembly processes of abundant and rare bacterial communities in paddy soils along the middle and lower reaches of the Yangtze River. We found that both abundant and rare bacterial communities exhibit diverse biogeographic patterns and are subject to different assembly processes, with abundant communities being more influenced by stochastic processes. Soil properties and geographic variables play pivotal roles in shaping the diversity and structure of both abundant and rare communities. Additionally, the predicted functions of abundant and rare communities differ, suggesting that rare communities may have a more significant impact on maintaining ecosystem functions. This study could enhance our understanding of the generation, maintenance, and driving mechanisms of soil bacterial biodiversity in agricultural ecosystems.

## AUTHOR CONTRIBUTIONS

**Xiancan Zhu:** Conceptualization (lead); funding acquisition (supporting); investigation (equal); supervision (lead); writing – original draft (equal); writing – review and editing (equal). **Minghui Hu:** Data curation (equal); investigation (equal); writing – original draft (equal). **Xiaoli Wang:** Formal analysis (equal); investigation (equal); software (equal). **Ya Zhang:** Methodology (equal); writing – original draft (equal). **Dongsheng Du:** Formal analysis (equal); writing – review and editing (equal).

## CONFLICT OF INTEREST STATEMENT

The authors declare no conflicts of interest.

## Supporting information


Appendix S1.

## Data Availability

The data that support the findings of this study are openly available in NCBI at https://www.ncbi.nlm.nih.gov/bioproject/PRJNA822031/.
