# Supplementary material for: Biogeography and diversity patterns of abundant and rare bacterial communities in paddy soils along middle and lower Yangtze River
Source: Ecol Evol. 2024 Jun 4;14(6):e11481. doi: 10.1002/ece3.11481 (PMC11148396; doi:10.1002/ece3.11481)
Supplement: Supplementary file 1 — Appendix S1. [file ECE3-14-e11481-s001.docx]

**Table S1** Diversity of abundant and rare communities at 43 sampling sites along the middle and lower reaches of the Yangtze River. The value were calculated from means of 3 samples at each sampling site

| Sampling sites | ASV richness |  | Shannon index |  |
| --- | --- | --- | --- | --- |
|  | Abundant | Rare | Abundant | Rare |
| Y1 | 18.33 | 2682 | 3.64 | 10.79 |
| Y2 | 22.20 | 2895 | 4.25 | 10.97 |
| Y3 | 18.57 | 3157 | 3.79 | 11.08 |
| Y4 | 17.63 | 2854 | 3.71 | 10.83 |
| Y5 | 17.63 | 2476 | 3.69 | 10.63 |
| Y6 | 21.27 | 2636 | 3.90 | 10.83 |
| Y7 | 21.90 | 2865 | 4.09 | 10.95 |
| Y8 | 22.30 | 2734 | 3.99 | 10.85 |
| Y9 | 22.97 | 2415 | 4.22 | 10.58 |
| Y10 | 9.33 | 2523 | 2.31 | 10.66 |
| Y11 | 24.20 | 2610 | 4.10 | 10.78 |
| Y12 | 18.67 | 2783 | 3.67 | 10.92 |
| Y13 | 23.87 | 2514 | 4.02 | 10.71 |
| Y14 | 22.43 | 2550 | 3.97 | 10.67 |
| Y15 | 23.30 | 2329 | 3.79 | 10.52 |
| Y16 | 24.27 | 2947 | 4.29 | 10.98 |
| Y17 | 23.23 | 2971 | 4.22 | 11.06 |
| Y18 | 19.13 | 3200 | 3.75 | 11.16 |
| Y19 | 26.87 | 2863 | 4.43 | 10.90 |
| Y20 | 13.93 | 2902 | 3.25 | 10.91 |
| Y21 | 27.53 | 2916 | 4.34 | 10.97 |
| Y22 | 16.23 | 2798 | 3.26 | 10.87 |
| Y23 | 19.23 | 2761 | 3.67 | 10.86 |
| Y24 | 20.67 | 2963 | 4.05 | 11.01 |
| Y25 | 27.30 | 2668 | 4.20 | 10.82 |
| Y26 | 22.87 | 2804 | 4.04 | 10.89 |
| Y27 | 16.23 | 2654 | 3.16 | 10.73 |
| Y28 | 22.80 | 2827 | 4.02 | 10.90 |
| Y29 | 13.90 | 2142 | 1.73 | 10.29 |
| Y30 | 15.27 | 2522 | 2.01 | 10.65 |
| Y31 | 16.13 | 2407 | 2.21 | 10.49 |
| Y32 | 24.03 | 2750 | 4.27 | 10.79 |
| Y33 | 21.33 | 2634 | 3.89 | 10.73 |
| Y34 | 9.33 | 2490 | 2.56 | 10.60 |
| Y35 | 21.67 | 2867 | 4.24 | 10.90 |
| Y36 | 20.90 | 2885 | 4.06 | 10.96 |
| Y37 | 23.67 | 3018 | 4.32 | 11.06 |
| Y38 | 19.63 | 3181 | 3.97 | 11.15 |
| Y39 | 17.67 | 2964 | 3.83 | 10.98 |
| Y40 | 19.23 | 3116 | 3.94 | 11.12 |
| Y41 | 21.00 | 2962 | 4.08 | 11.02 |
| Y42 | 19.33 | 2830 | 3.82 | .10.87 |
| Y43 | 18.57 | 2725 | 3.66 | 10.78 |

**Table S2** The correlation (r^2^) and significance (*P*) values of pairwise regressions between diversity of abundant and rare communities and geographic and soil variables by Spearman’s correlation coefficient.

| Variables | ASV richness | | | | Shannon index | | | |
| --- | --- | --- | --- | --- | --- | --- | --- | --- |
|  | Abundant | | Rare | | Abundant | | Rare | |
|  | r^2^ | *P* | r^2^ | *P* | r^2^ | *P* | r^2^ | *P* |
| Longitude | -0.106 | 0.231 | **0.201** | **0.022** | -0.033 | 0.709 | 0.157 | 0.075 |
| Latitude | **-0.194** | **0.028** | 0.113 | 0.201 | -0.107 | 0.227 | 0.055 | 0.535 |
| pH | **0.278** | **0.001** | 0.037 | 0.681 | **0.324** | **0.000** | 0.070 | 0.428 |
| SOM | **0.195** | **0.027** | **0.205** | **0.020** | **0.293** | **0.001** | 0.171 | 0.053 |
| Total N | **0.223** | **0.011** | **0.174** | **0.048** | **0.272** | **0.002** | 0.146 | 0.099 |
| Olsen-P | **0.185** | **0.036** | 0.061 | 0.491 | 0.221 | 0.012 | 0.057 | 0.523 |
| NH_4_^+^-N | -0.033 | 0.708 | -0.115 | 0.196 | -0.082 | 0.355 | -0.150 | 0.089 |
| NO_3_^-^-N | 0.133 | 0.134 | **0.219** | **0.013** | **0.227** | **0.010** | 0.162 | 0.067 |
| Urease | **0.570** | **0.000** | 0.070 | 0.432 | **0.570** | **0.000** | 0.111 | 0.209 |
| Invertase | **0.249** | **0.004** | 0.151 | 0.088 | **0.252** | **0.004** | 0.128 | 0.148 |
| Phosphatase | -0.023 | 0.792 | **0.188** | **0.032** | 0.002 | 0.980 | 0.108 | 0.222 |

SOM, soil organic matter.

**Table S3** The correlation (r^2^) and significance (*P*) values of pairwise regressions between PCoA scores of abundant and rare communities and geographic and soil variables.

| Variables | PCoA1 | | | | PCoA2 | | | |
| --- | --- | --- | --- | --- | --- | --- | --- | --- |
|  | Abundant | | Rare | | Abundant | | Rare | |
|  | r^2^ | *P* | r^2^ | *P* | r^2^ | *P* | r^2^ | *P* |
| Longitude | -0.126 | 0.155 | 0.008 | 0.928 | **-0.365** | **0.000** | **0.179** | **0.043** |
| Latitude | -0.028 | 0.753 | -0.029 | 0.747 | **-0.328** | **0.000** | 0.072 | 0.419 |
| pH | **-0.507** | **0.000** | **0.495** | **0.000** | **-0.322** | **0.000** | 0.028 | 0.755 |
| SOM | -0.063 | 0.477 | **0.203** | **0.021** | **0.427** | **0.000** | **-0.352** | **0.000** |
| Total N | -0.102 | 0.251 | **0.262** | **0.003** | **0.361** | **0.000** | **-0.314** | **0.000** |
| Olsen-P | 0.101 | 0.254 | -0.109 | 0.217 | 0.088 | 0.321 | 0.028 | 0.757 |
| NH_4_^+^-N | **0.329** | **0.000** | **-0.369** | **0.000** | 0.022 | 0.808 | **0.245** | **0.005** |
| NO_3_^-^-N | **-0.446** | **0.000** | **0.448** | **0.000** | -0.157 | 0.076 | **-0.204** | **0.021** |
| Urease | **-0.701** | **0.000** | **0.605** | **0.000** | **-0.372** | **0.000** | 0.164 | 0.063 |
| Invertase | **-0.218** | **0.013** | **0.335** | **0.000** | **0.177** | **0.045** | **-0.279** | **0.001** |
| Phosphatase | 0.025 | 0.780 | 0.073 | 0.410 | 0.078 | 0.381 | **-0.290** | **0.001** |

SOM, soil organic matter.

**Table S4** The correlation (r^2^) and significance (*P*) values of pairwise regressions between PCoA scores of abundant and rare community functional structure and geographic and soil variables.

| Variables | PCoA1 | | | | PCoA2 | | | |
| --- | --- | --- | --- | --- | --- | --- | --- | --- |
|  | Abundant | | Rare | | Abundant | | Rare | |
|  | r^2^ | *P* | r^2^ | *P* | r^2^ | *P* | r^2^ | *P* |
| Longitude | **-0.270** | **0.002** | **0.352** | **0.000** | **-0.187** | **0.034** | **-0.175** | **0.047** |
| Latitude | **-0.310** | **0.000** | **0.355** | **0.000** | **-0.295** | **0.001** | -0.097 | 0.276 |
| pH | -0.156 | 0.077 | -0.144 | 0.102 | **0.215** | **0.014** | **-0.454** | **0.000** |
| SOM | **0.551** | **0.000** | **-0.445** | **0.000** | -0.066 | 0.456 | **0.373** | **0.000** |
| Total N | **0.523** | **0.000** | **-0.469** | **0.000** | 0.061 | 0.491 | **0.349** | **0.000** |
| Olsen-P | **0.180** | **0.042** | -0.019 | 0.828 | **-0.215** | **0.014** | **0.293** | **0.001** |
| NH_4_^+^-N | -0.090 | 0.311 | 0.170 | 0.054 | -0.148 | 0.094 | **0.304** | **0.000** |
| NO_3_^-^-N | -0.098 | 0.269 | 0.056 | 0.531 | -0.153 | 0.083 | 0.012 | 0.896 |
| Urease | 0.110 | 0.215 | **-0.176** | **0.045** | **0.300** | **0.001** | -0.003 | 0.977 |
| Invertase | **0.412** | **0.000** | **-0.247** | **0.005** | 0.077 | 0.386 | **0.228** | **0.009** |
| Phosphatase | 0.142 | 0.108 | -0.043 | 0.632 | -0.089 | 0.318 | **0.224** | **0.011** |

SOM, soil organic matter.

**
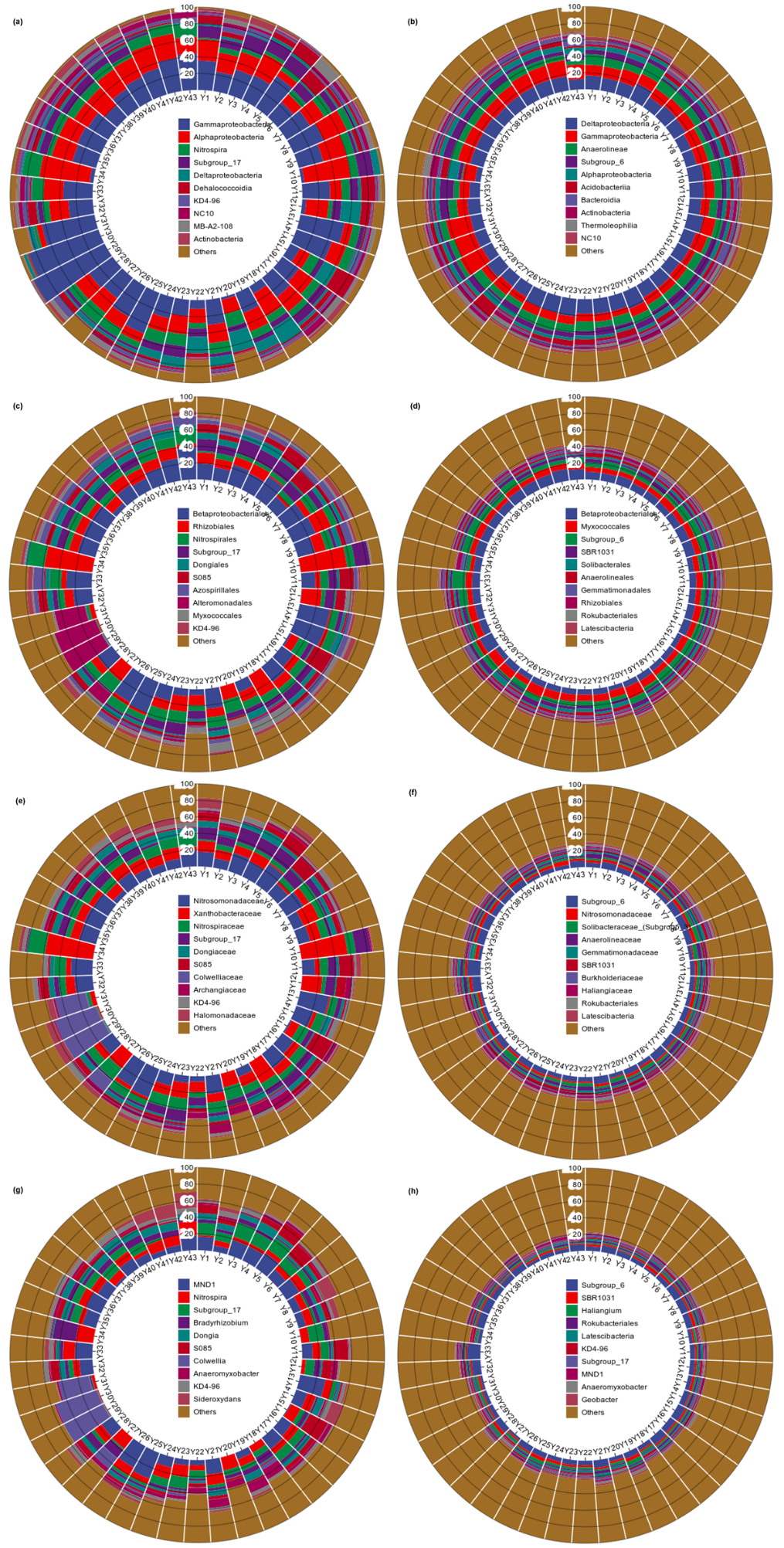
**

**Fig. S1** Relative abundances of top 10 taxonomic abundant (a, class; c, order; e, family; g, genus) and rare (b, class; d, order; f, family; h, genus) communities at 43 sampling sites.


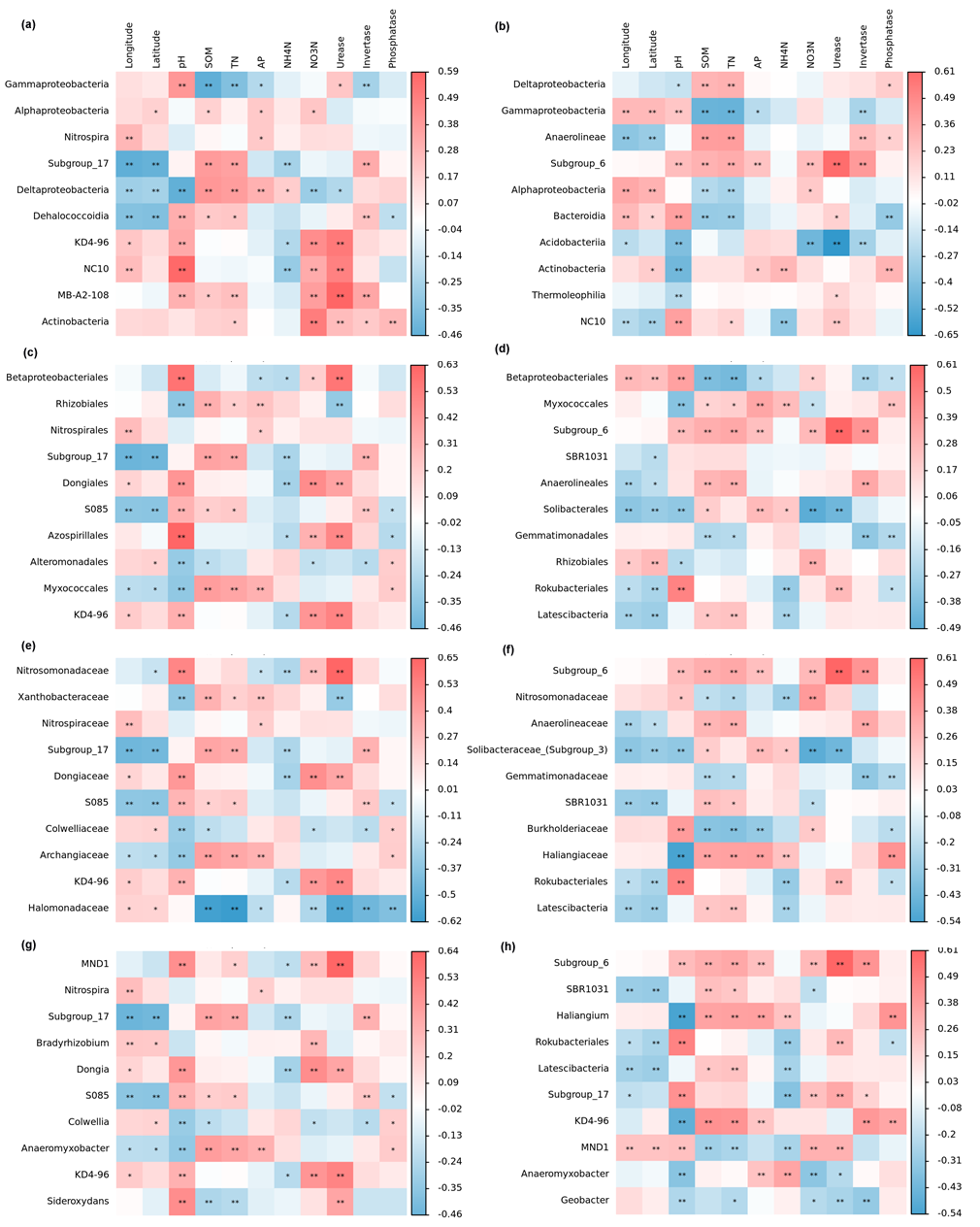


**Fig. S2** Relationships of relative abundances of top 10 taxonomic abundant (a, class; c, order; e, family; g, genus) and rare (b, class; d, order; f, family; h, genus) communities with geographic and soil variables.

**Fig. S3** Structures of abundant and rare communities analyzed through PCoA plots across diverse sites


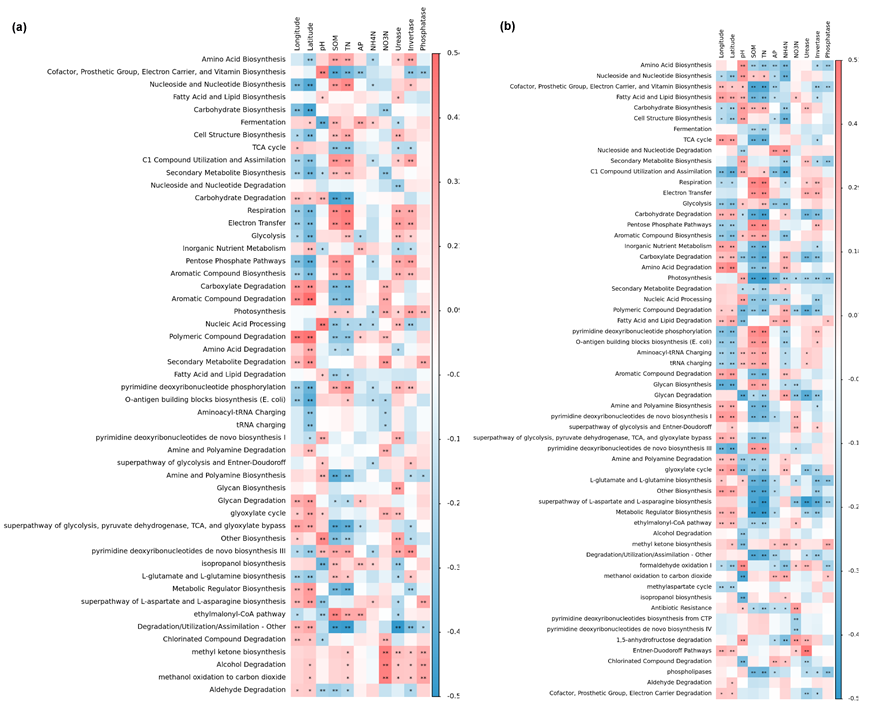


**Fig. S4** Relationship between predicted KEEG functional abundances of abundant (a) and rare (b) communities and geographic and soil variables.
